# Incidental hyperglycemia and myocardial infarction risk in non-diabetic patients in the emergency department: A retrospective cohort analysis

**DOI:** 10.2478/jccm-2025-0033

**Published:** 2025-10-31

**Authors:** Erkan Boğa

**Affiliations:** Ministry of Health of Turkey, Istanbul, Turkey

**Keywords:** hyperglycemia, myocardial infarction, non-diabetic patients, cardiovascular risk, emergency department

## Abstract

**Objective:**

This study investigated whether incidental hyperglycemia serves as an independent risk factor for myocardial infarction (MI) among non-diabetic patients in the emergency department.

**Methods:**

A retrospective case-control study analyzed data from one thousand non-diabetic patients aged 18–85 years who visited the emergency department during January through October two thousand twenty-four Patients were classified into two equal groups based on their random blood glucose levels: patients with glucose levels above 140 mg/dL formed the hyperglycemia group and patients with glucose levels below 140 mg/dL belonged to the normoglycemia group. The analysis employed logistic regression to assess how hyperglycemia related to MI while controlling for various demographic and clinical variables.

**Results:**

The incidence of MI was found in 61.4% of patients with hyperglycemia but only in 25.8% of patients with normoglycemia. Multivariable analysis revealed that incidental hyperglycemia increased the odds of MI by 2.42 times. The risk was higher among male patients and further increased when glucose levels exceeded 180 mg/dL.

**Conclusions:**

Non-diabetic emergency department patients who experience incidental hyperglycemia show a high risk of developing MI. The evaluation of cardiovascular risk should begin with emergency physicians, who should consider elevated random blood glucose as a potential marker for identifying patients likely to benefit from early assessment and follow-up.

## İntroduction

Hyperglycemia is commonly associated with diabetes mellitus but may also occur in non-diabetic individuals under acute stress. Stress-induced hyperglycemia is defined as a transient elevation in blood glucose levels in response to acute illness, injury, surgery, neurological conditions, or cardiovascular events [1]. During acute stress, hormones such as catecholamines, cortisol, growth hormone, and glucagon are released, stimulating hepatic gluconeogenesis and glycogenolysis, which leads to increased plasma glucose levels [2].

The prognostic value of stress-induced hyperglycemia in patients with cardiovascular diseases, including acute coronary syndromes (ACS), has been extensively studied. Myocardial infarction (MI) is one of the most prevalent and severe medical conditions worldwide [3]. The pathophysiological mechanisms of MI include atherosclerotic plaque rupture, endothelial dysfunction, inflammation, thrombosis, and microvascular injury [4]. Hyperglycemia is thought to increase the risk of MI by exacerbating endothelial dysfunction, activating oxidative stress pathways, and promoting thrombosis [5].

Previous studies have shown that acute stress-induced hyperglycemia may be associated with poorer clinical outcomes in non-diabetic individuals [6]. For instance, in patients with acute coronary syndrome, the association between hyperglycemia and mortality is well-documented [7]. However, the number of studies investigating the relationship between incidental hyperglycemia assessed in the emergency department (ED) and MI occurrence in non-diabetic patients is limited. If elevated random blood glucose levels in the ED are found to be an independent risk factor for cardiovascular events, this could be important for early diagnosis and management by emergency physicians.

Current literature suggests that hyperglycemia is a risk factor for cardiovascular events not only in diabetic patients but also in those without diabetes [8]. In particular, hyperglycemia under acute stress conditions may exacerbate inflammation, impair vascular function, and promote a prothrombotic state in non-diabetic individuals [9]. This mechanism is linked directly to MI via plaque instability, thrombus formation and oxidative stress [10].

Several studies have reported that hyperglycemia during hospitalization increases post-MI mortality rates [11]. However, the association between incidental hyperglycemia measured in the emergency department and MI risk has not been thoroughly explored. It is therefore essential to determine whether incidental hyperglycemia in non-diabetic patients presenting to the emergency department serves as a predictor of MI [12]. Early identification of this risk may prevent diagnostic delays and improve management. If confirmed, these findings may warrant updates to cardiovascular risk classification systems used in emergency settings [13].

The purpose of this paper is to determine the clinical importance of incidental hyperglycemia in non-diabetic patients in the emergency department and to find out if it can be used as a risk indicator by emergency physicians [14].

## Methods

This observational case-control study employed a retrospective design. The primary objective was to determine whether incidental hyperglycemia during emergency department visits in non-diabetic patients serves as an independent predictor of MI.

A 1:1 matching method was used to create two groups: the hyperglycemia group (≥140 mg/dL, n=500) and the normoglycemia (control) group (<140 mg/dL, n=500). These groups were compared to assess the association between hyperglycemia and MI.

The study was conducted in the Emergency Department of Esenyurt Necmi Kadıoğlu State Hospital and included patients who presented between January 1 and October 31, 2024. The patient records were reviewed in retrospect, and the data were collected from the hospital information management system.

**Study Population and Inclusion Criteria:** Eligible participants were aged between 18 and 85 years. Inclusion required presentation to the emergency department, a random blood glucose test, and evaluation for MI via ECG and troponin T testing.

**Exclusion Criteria:** Patients with a prior diagnosis of diabetes mellitus (HbA1c ≥6.5% or fasting blood glucose ≥126 mg/dL), those using corticosteroids or other glucose-raising medications, and patients with chronic kidney disease, cirrhosis, or endocrine disorders were excluded. Incomplete or missing data also led to exclusion.

Patients were matched 1:1 based on age (±5 years), gender, hypertension, hyperlipidemia, and smoking status, resulting in two equal groups. This matching strategy aimed to minimize confounding and allow a more accurate comparison of MI risk.

This matching method was intended to reduce the impact of confounding factors and to offer a more accurate comparison of MI risk between hyperglycemic and normoglycemic patients.

**Dependent Variable:** MI diagnosis. The diagnosis was made according to American Heart Association criteria using troponin T and ECG.

**Independent Variable:** An incidental blood glucose level (mg/dL) was measured in the emergency department. Hyperglycemia was defined as a glucose level ≥140 mg/dL.

**Potential Confounders:** Age, gender, hypertension, hyperlipidemia, smoking status, family history of early cardiovascular disease, and body mass index (BMI).

**Laboratory Measurements:** Blood glucose levels were assessed with the Roche Accu-Chek device, and troponin T was assessed with the Roche Elecsys Troponin T high-sensitive assay.

**Strategies to Minimize Bias:** As this study is retrospective, there are some risks of bias. To minimize these risks, a case-control design was used, and the case and control groups were matched 1:1 by baseline characteristics. Incomplete data were reviewed and corrected before analysis. Data validation was done by two independent researchers to assure the accuracy of the records.

**Descriptive statistics:** Continuous variables were presented as mean ± standard deviation (SD). Categorical variables were presented as number (n) and percentage (%).

**Comparisons between groups:** Paired t-test or Wilcoxon signed-rank test for continuous variables. Mc-Nemar’s test for categorical variables.

**Assessment of hyperglycemia-MI relationship:** The association between hyperglycemia and MI was examined using logistic regression analysis. Multivariate analysis was performed to include simultaneous control of potential confounders, including age, gender, hypertension, smoking, and hyperlipidemia.

**Missing Data:** Data with missing values <5% were ignored. In case of missing data >5%, multiple imputation was employed for data completion.

**Subgroup and Sensitivity Analyses:** The association between hyperglycemia and MI was explored within distinct age and gender subgroups. Hyperglycemia was defined at different thresholds (≥160 and ≥180 mg/dL) to examine the difference in MI risk in sensitivity analyses.

**Sample size and power calculation:** The sample size was calculated to provide 80% statistical power with a 5% margin of error. In order to detect the anticipated difference in MI incidence, power analysis suggested that at least 500 patients were needed per group. One thousand patients were recruited in the study.

### Ethical Approval

This study was conducted in accordance with the ethical standards of the Declaration of Helsinki and approved by the Non-Interventional Clinical Research Ethics Committee of Istanbul Medipol University. The research protocol titled *“Incidental Hyperglycemia and Myocardial Infarction Risk in Non-Diabetic Patients in the Emergency Department: A Retrospective Cohort Analysis”* was reviewed and approved with the decision number 290, dated March 6, 2025. As this was a retrospective study using anonymized patient data obtained from hospital records, informed consent was not required.

## Results

A total of 1,200 patients were initially screened. After applying the inclusion and exclusion criteria, 1,000 patients were included in the final analysis. Of the 200 excluded patients, 120 had a prior diagnosis of diabetes mellitus, and 80 had incomplete or erroneous data.

The 1,000 patients were divided into two groups in a 1:1 ratio based on the predefined criteria: the Hyperglycemia Group (n=500), with random blood glucose ≥140 mg/dL, and the Control Group (n=500), with glucose <140 mg/dL (Table 1).

**Table 1.** Patient Characteristics

**Parameter**	**Hyperglycemia Group (≥140 mg/dL)**	**Control Group (<140 mg/dL)**
Mean Age (years)	60.3 ± 10.4	58.7 ± 11.2
Gender Distribution	Female 48.5%, Male 51.5%	Female 48.5%, Male 51.5%
Hypertension Prevalence	45.6%	39.8%
Hyperlipidemia Prevalence	50.2%	44.1%
Smoking Status	28.4%	24.7%
BMI (kg/m^2^)	27.8 ± 5.1	27.8 ± 5.1
Random Blood Glucose (mg/dL)	160.2 ± 18.3	101.7 ± 12.6
Troponin T Level (ng/mL)	0.74 ± 0.42	0.18 ± 0.11

As a retrospective study, the analysis was based on the biochemical and clinical data at the time of hospital admission, and not on follow-up data.

The demographic, clinical, and biochemical characteristics of the patients included in the study are presented below.

No variable had more than 5% missing data, as determined by missing data analysis.

**Fig. 1.**
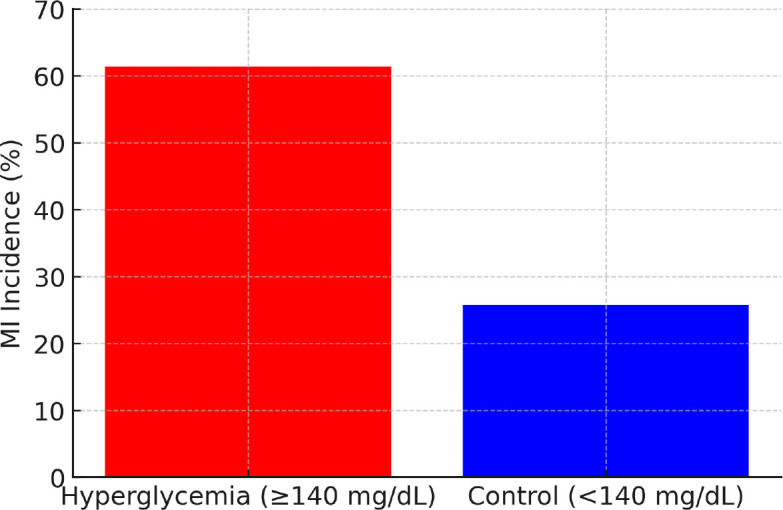
Incidence of Myocardial Infarction

Overall, 436 out of 1,000 patients (43.6%) were diagnosed with MI. The incidence of MI differed significantly between groups: 61.4% (n=307) in the Hyperglycemia Group versus 25.8% (n=129) in the Control Group (p < 0.001; Figure 1).

Logistic regression analysis revealed that hyperglycemia was a significant predictor of MI, increasing the risk by 2.7 times (p < 0.001; Table 2).

**Table 2.** Logistic Regression Analysis

**Variable**	**Odds Ratio (OR)**	**95% Confidence Interval (CI)**	**p-value**
Hyperglycemia	2.42	1.98 – 2.95	<0.001
Age	1.05	1.02 – 1.08	0.004
Hypertension	1.48	1.21 – 1.83	0.001
Smoking	1.56	1.27 – 1.91	0.002

After adjusting for potential confounders—including age, gender, hypertension, hyperlipidemia, and smoking—hyperglycemia remained a significant independent predictor of MI.

These findings indicate that hyperglycemia is an independent predictor of MI.

Subgroup Analysis by Gender: The association between hyperglycemia and MI was stronger in male patients (OR: 2.65, p < 0.001), although it remained significant in female patients (OR: 2.21, p = 0.003).

**Fig. 2.**
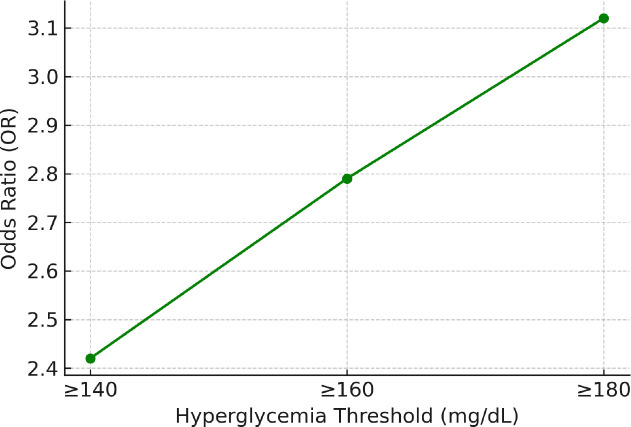
MI Risk Based on Hyperglycemia Severity

MI risk based on hyperglycemia severity: When the hyperglycemia threshold was increased to ≥180 mg/dL, the risk of MI rose further (OR: 3.12, 95% CI: 2.45–3.98, p < 0.001). A significant association was also observed at ≥160 mg/dL (OR: 2.79, p < 0.001). These findings indicate a dose-response relationship between hyperglycemia severity and MI risk (Figure 2).

## Discussion

This study assessed whether incidental hyperglycemia detected in the emergency department could serve as an independent risk factor for MI in non-diabetic patients. The results demonstrated that patients with hyperglycemia had a significantly higher incidence of MI compared to those with normoglycemia (61.4% vs. 25.8%, p < 0.001). Logistic regression analysis revealed a 2.4-fold increase in MI risk associated with hyperglycemia, independent of age, sex, hypertension, hyperlipidemia, and smoking status. The risk of MI increased proportionally with higher blood glucose levels, reaching 3.12 times above baseline when glucose exceeded 180 mg/dL (p < 0.001).

Subgroup analysis revealed that the association between hyperglycemia and MI was stronger in male patients (OR: 2.65), but remained statistically significant in female patients as well (OR: 2.21). These findings support the role of stress-induced hyperglycemia as a relevant biomarker for cardiovascular risk in patients without a diabetes diagnosis. The results align with previous studies demonstrating the predictive value of admission blood glucose levels in acute coronary syndromes. This study differs from previous research by specifically analyzing incidental hyperglycemia in non-diabetic patients presenting to the emergency department, thereby offering unique insights into risk stratification for this population.

These findings suggest that incidental hyperglycemia should be considered a potential marker of underlying cardiovascular disease rather than merely a transient stress response. Random glucose testing in the emergency department may provide valuable information not only for identifying undiagnosed diabetes but also for early cardiovascular risk assessment. Clinical evaluation of non-diabetic patients with elevated glucose levels should include cardiovascular risk screening and long-term follow-up. To implement these findings in clinical practice, further research is needed to define optimal glucose thresholds for risk stratification, evaluate long-term outcomes, and develop standardized protocols for initial management in emergency settings.

### Limitations

This study has several limitations. Its retrospective case-control design limits the ability to establish causal relationships and introduces the potential for selection bias and inaccuracies in historical records. Additionally, the study was conducted at a single center with a specific patient population and institutional protocols, which may limit the generalizability of the findings. The use of random blood glucose measurements to define hyperglycemia is another limitation, as these values may fluctuate in response to acute stress and may not reflect true baseline glycemic status. Although sensitivity analyses using different glucose thresholds confirmed the primary findings, future studies should consider using standardized glycemic markers. The analysis did not account for several potential confounding factors, such as undiagnosed diabetes, inflammatory markers, medications, and socioeconomic variables. The study did not differentiate between ST-elevation myocardial infarction (STEMI) and non-ST-elevation myocardial infarction (NSTEMI) subtypes. Additionally, it lacked information on diagnostic procedures and therapeutic interventions, such as coronary angiography and percutaneous coronary intervention (PCI). The absence of long-term follow-up data limits the ability to assess persistent cardiovascular risk after hospital discharge.

## Conclusion

This study demonstrated that hyperglycemia detected in the emergency department is an independent risk factor for MI in non-diabetic patients. Patients with elevated blood glucose levels had significantly higher odds of MI, and this association remained significant after adjusting for traditional cardiovascular risk factors such as age, gender, hypertension, hyperlipidemia, and smoking. Moreover, the risk of MI increased proportionally with the severity of hyperglycemia. These findings suggest that emergency physicians should consider elevated random blood glucose as a relevant marker in cardiovascular risk assessment. Further large-scale, prospective, multicenter studies are needed to confirm these results and guide clinical practice.
